# *Gleditsia sinensis*: Transcriptome Sequencing, Construction, and Application of Its Protein-Protein Interaction Network

**DOI:** 10.1155/2014/404578

**Published:** 2014-05-27

**Authors:** Liucun Zhu, Ying Zhang, Wenna Guo, Qiang Wang

**Affiliations:** ^1^Institute of System Biology, Shanghai University, Shanghai 200444, China; ^2^Yangzhou Breeding Biological Agriculture Technology Co. Ltd., Yangzhou 225200, China; ^3^State Key Laboratory of Pharmaceutical Biotechnology, School of Life Sciences, Nanjing University, Nanjing 210093, China

## Abstract

*Gleditsia sinensis* is a genus of deciduous tree in the family *Caesalpinioideae*, native to China, and is of great economic importance. However, despite its economic value, gene sequence information is strongly lacking. In the present study, transcriptome sequencing of *G. sinensis* was performed resulting in approximately 75.5 million clean reads assembled into 142155 unique transcripts generating 58583 unigenes. The average length of the unigenes was 900 bp, with an N50 of 549 bp. The obtained unigene sequences were then compared to four protein databases to include NCBI nonredundant protein (NRDB), Swiss-prot, Kyoto Encyclopedia of Genes and Genomes (KEGG), and the Cluster of Orthologous Groups (COG). Using BLAST procedure, 31385 unigenes (53.6%) were generated to have functional annotations. Additionally, sequence homologies between identified unigenes and genes of known species in a protein-protein interaction (PPI) network facilitated *G. sinensis* PPI network construction. Based on this network construction, new stress resistance genes (including cold, drought, and high salinity) were predicted. The present study is the first investigation of genome-wide gene expression in *G. sinensis* with the results providing a basis for future functional genomic studies relating to this species.

## 1. Introduction

*Gleditsia sinensis* is a genus of deciduous tree in the family Caesalpinioideae, native to China.* G*.* sinensis* usually grows 15–30 m tall and is of economic and medicinal importance. The fruits of* G. sinensis* can serve as medicine, food, health products, cosmetics, and natural raw materials for cleaning products [1]; the seeds can be used as appetizing medicine [2, 3] and contain an important vegetable gum (guar gum) [2]; the thorns (soap-pin) contain flavonoid glycosides, phenols, and amino acids and have a high economic value [3]. However, up to October 2013, only 17 nucleotide sequences and eight protein sequences of* G. sinensis* were available in the NCBI database. This brings to question why such an economically valuable organism has been so understudied, making it important to generate more genetic sequence information to further study* G. sinensis*.

Plants are exposed to continuously changing environmental conditions under natural conditions. Various environmental stresses, such as heat, cold, drought, and high salinity, are major factors in affecting plant development, growth, and productivity [4–6]. The stress-induced transcriptomic responses of plants revealed that many molecular mechanisms had been evolved to help plants to adapt and survive under the harmful stresses. Usually, there is an initial activation/sensory stage followed by a physiological stage when the plant perceived and responded to the abiotic stress [6, 7]. Previous work in a variety of stresses has been studied in* Arabidopsis thaliana *[8, 9]. Compared to* Arabidopsis thaliana*, there is little known about how trees respond to abiotic stress. In recent years, the emergence of next-generation sequencing technology has provided a fast and effective approach to generation of transcriptome data of nonmodel organisms lacking a complete genome sequence [10, 11]. Compared with whole-genome sequencing, RNA sequencing (RNA-seq) is of low cost and high throughput, becoming an important part of functional genome research [12]. It provided an efficient way of identifying the expression level and new members of the genes [13, 14].

In the present study, the Illumina HiSeq 2000 platform was used for transcriptome sequencing in four tissue types collected from* G. sinensis*. A total of 7632619288 reads assembled into 58583 unigenes and their functional annotations were obtained. In addition, a protein-protein interaction (PPI) network comprising genes expressed in* G. sinensis* was constructed and utilized to identify some new potential drought, freezing, and salinity tolerance genes. These findings will provide a solid foundation for further investigation of the functional genomics of* G. sinensis*.

## 2. Materials and Method

### 2.1. RNA Preparation, Sequencing, and Assembly

The* G. sinensis* specimen used in the present study was wild-grown from the Jiangsu Province, China. Total RNA was extracted from four tissues: tender shoots, young leaves, flower buds, and round thorns, using TRIzol Reagent with qualification and quantification evaluated by Agilent 2100 Bioanalyzer Nanochips and NanoDrop 2000 Spectrophotometer. And then it was processed and used for Illumina sequencing [15].

Raw read sequences are uploaded in the Short Read Archive database from National Center for Biotechnology Information (NCBI) with the accession number SRR 1012862.

We used SeqPreq (https://github.com/jstjohn/SeqPrep) to remove sequencing adapters and then used sickle [16] to trim low-quality sequences with default parameters. After cleaning reads, all of the high-quality reads were used in assembling by Trinity (trinityrnaseq_r2013-02-25) [17, 18]. The k-mer was counted by jellyfish and the min_contig_length was set as 300. Then, RSEM [19] was used to measure expression levels of every unique transcript. Units of TPM (transcripts per million) were used to report results. After counting fraction of each isoform, length × isoform percent was defined as a standard to identify unigenes.

### 2.2. Functional Annotation

Unigenes larger than 300 bp were subjected to functional annotation for predicting putative gene descriptions, conserved domains, gene ontology (GO) terms, and association with metabolic pathways. First, BLAST procedure (ftp://ftp.ncbi.nih.gov/blast/executables/release/2.2.18/) was used to compare all unigenes against protein sequence databases to include NRDB, Swiss-prot, and Clusters of Orthologous Groups (COG) with an *E*-value < 1.0*E* − 6. Based on BLAST best match results, gene function and protein-related information were predicted, with Blast2GO software [20] used for gene ontology (GO) annotation in terms of molecular function, cellular component, and biological process. After all GO annotations were obtained, Web Gene Ontology Annotation Plot (WEGO) software was used to construct comparative plots based on unigene classifications. Unigenes were also subjected to sequence comparison using the COG database for gene function prediction [21]. For Kyoto Encyclopedia of Genes and Genomes (KEGG) annotation [22], the BLASTX software was used for sequence comparison of unigenes within the KEGG database and completed on the KASS website [23] (http://www.genome.jp/tools/kaas/). Following KEGG annotation, each gene acquired a KEGG Orthology (KO) number representing a node in a certain reference metabolic pathway in KEGG.

### 2.3. Construction and Analysis of PPI Networks

First, known PPI networks and protein sequences from six species (*Arabidopsis thaliana*,* Arabidopsis lyrata*,* Oryza sativa* subsp.* japonica*,* Brachypodium distachyon*,* Populus trichocarpa*, and* Sorghum bicolor*) were downloaded from the String database [24]. Then using the TBLASTN software, protein sequences from the downloaded PPIs were compared with* G. sinensis* unigenes to identify homologous sequences with an *E*-value < 1.0*E* − 6. The criterion of candidate interacting genes of the network was the TBLASTN hits with identity >50% and covering query gene >80%. If two unigenes from* G. sinensis* corresponded to two homologous proteins in the known networks, the encoded proteins were considered to interact with each other. Concluding network construction, each node in the network was assigned to a degree *k*, which is the number of connected neighboring nodes. The degree distribution of giant network branches was computed using the formula *P*(*k*) = *N*(*k*)/*N* where *N* is the number of nodes and *N*(*k*) denotes the number of nodes with degree *k* [25].

Stress resistance genes and protein sequences of* Arabidopsis thaliana* proteins related to salinity, drought, and freezing tolerance were downloaded from the stress responsive transcription factor database [26–28] (STIFDB; http://caps.ncbs.res.in/stifdb2/) and compared with the* G. sinensis* unigene library to search for homologous sequences potentially possessing the same functions in* G. sinensis*. Next, the PPI network was used to predict novel drought, freezing, and salinity tolerance genes. The specific predictive criterions are as follows. If a protein in the* G. sinensis* network connects directly with the homologous sequences of over four known stress resistance genes, with no homology among these genes, then that gene was predicted to be a potential stress resistance gene.

## 3. Result

### 3.1. Paired-End Sequencing and* De Novo* Assembly of* G. sinensis* Transcriptome

**Figure 1:**
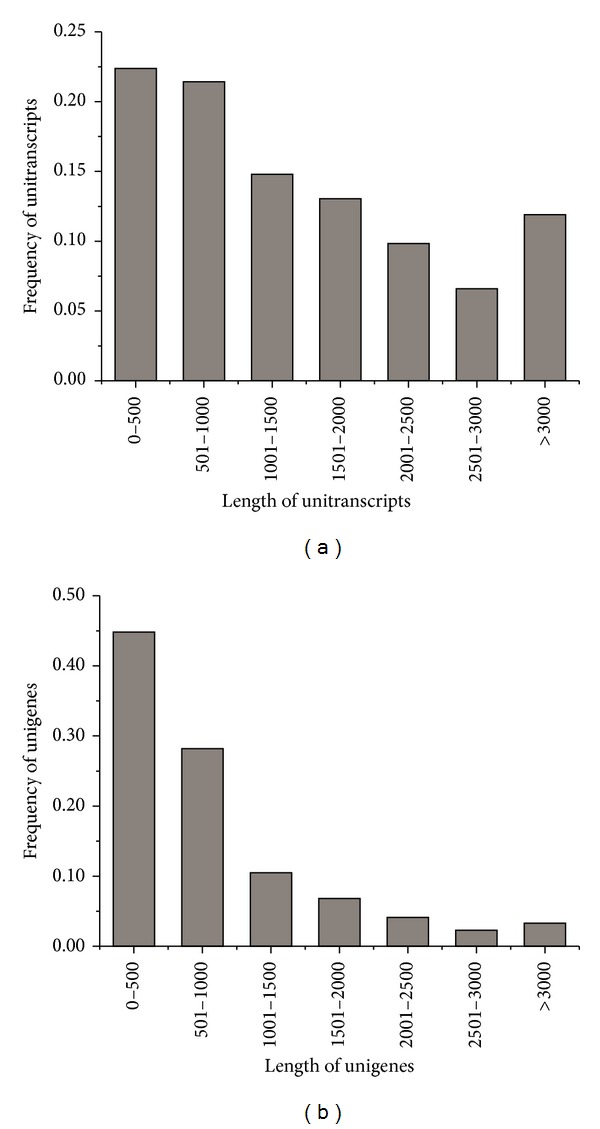
Overview of the* G. sinensis* transcriptome assembly. The size distribution of the UTs (a) and unigenes (b) produced from* de novo* assembly of reads by Trinity.

Genes are differentially expressed in different tissue types. In an effort to broaden the obtained gene expression profile in* G. sinensis*, total RNA was extracted from four different tissues (tender shoots, young leaves, flower buds, and round thorns) and mixed in equal parts for sequencing using the Illumina platform. This resulted in a total of 75.6 million high-quality clean reads containing 7632619288 nucleotides (nt) and an average read length of 101 bp (Table 1). Due to a lack of* G. sinensis* whole-genome sequence, Trinity software was used to assemble all high-quality clean reads into transcripts* de novo*. From all of the clean reads, a total of 142155 unique transcripts with an average length of 1537 bp were obtained with a N50 of 1202 bp and the majority of unique transcripts (31818) between 100 and 500 bp (Figure 1(a)). Then after removing redundant sequences, 58583 unigenes were obtained, with an average length of 900 bp. The lengths of the unigenes varied from 300 bp to over 3000 bp and the length distribution of unigenes was shown in Figure 1(b).

### 3.2. Functional Annotation and Classification of* G. sinensis* Transcriptome

Nonredundant database (NRDB) built in NCBI contains large amounts of protein information. For annotation of the* G. sinensis* transcriptome, all unigenes were compared against the NRDB using BLASTX (an *E*-value cut-off of 1*e* ^−6^) to reveal 31100 unigenes with sequence homology. Among them, 45.1% of unigenes have the best matches mapping soybean.

**Figure 2:**
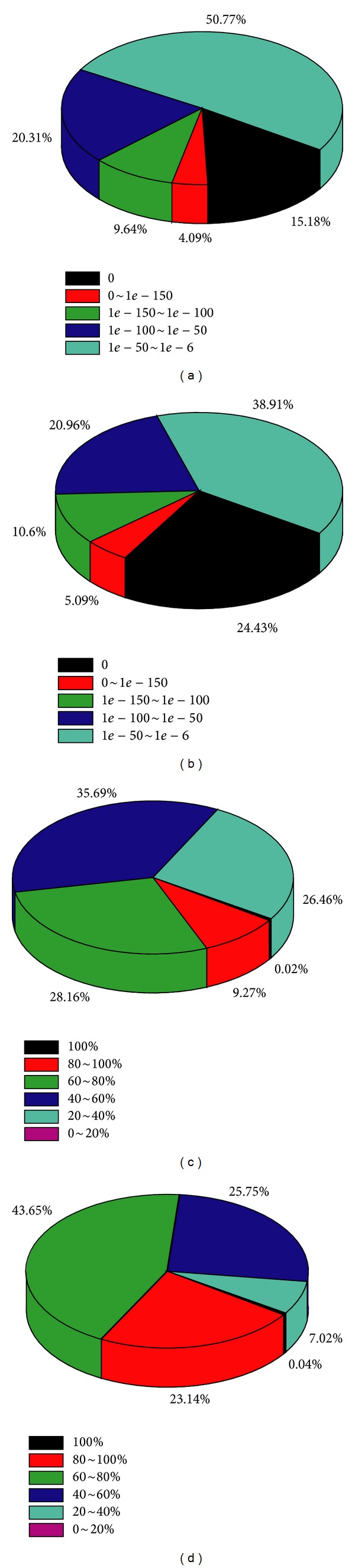
Unigene homology searches against NR and Swiss-prot databases shown by pie graphs. *E*-value proportional frequency distribution of BLAST hits against the NR database (a) and Swiss-prot database (c). Proportional frequency distribution of UTs similarities against the NR database (b) and Swiss-prot database (d) based on the best BLAST hits (*E*-value ≤ 1.0*E* − 6).

The distribution of *E*-values based on sequence homology showed that 61.1% of unigenes had high homology (smaller than 1.0*e* − 50) with the *E*-values of the other matches varying from 1.0*E* − 50 to 1.0*E* − 6 (Figure 2(a)). The similarity distribution showed the majority of unigenes (93.0%) with homologous sequences having similarities between 40% and 100%, with only 7% of the unigenes with homologous sequences having similarities less than 40% (Figure 2(b)).

Swiss-prot, an annotated protein sequence database maintained by the European Bioinformatics Institute (EBI), was also employed for unigene comparison which revealed 22157 of 58583 unigenes (37.8%) with sequence homology at an *E*-value threshold of ≤1.0*E* − 6 (Table 2). Almost half of these unigenes (49.2%) had an *E*-value ≤ 1.0*E* − 50, and the remaining had *E*-values between 1.0*E* − 50 and 1.0*E* − 6 which showed a slightly different result compared to the NRDB query (Figure 2(c)). In Swiss-prot comparison, 73.4% of the unigenes had sequence homology in the range of 40% to 100%, with only 26.6% having homology <40% (Figure 2(d)). In short, when combining the results from Swiss-prot and NRDB comparisons, a total of 31131 unigenes were confirmed to have homologous sequences.

### 3.3. Classification by Gene Ontology (GO) Annotation

**Figure 3:**
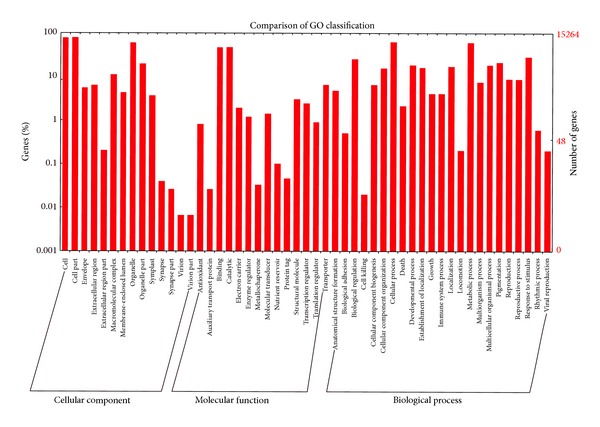
Gene ontology classification of the* G. sinensis* transcriptome. Gene ontology (GO) term assignments to* G. sinensis* unigenes based on significant hits against the NR database are summarized into three main GO categories (biological process, cellular component, and molecular function) and 51 subcategories.

Gene ontology (GO) terms were utilized to assign gene function classifications to each unigene based on sequence similarity comparisons from NRDB. Among the 58583 unigenes identified in* G. sinensis*, 15264 were categorized into at least one of the three main GO categories which could be further subdivided into 51 subcategories (Table 2, Figure 3, and Additional File 1; see Supplementary Material available online at http://dx.doi.org/10.1155/2014/404578). The number of unigenes in cellular component, biological process, and molecular function was 11942 (20.4%), 11683 (19.9%), and 11129 (19.0%), respectively. The subcategory at the “cell,” “cell part,” “cellular process,” “organelle,” “metabolic process,” “binding,” and “catalytic activity” level included the highest number of unigenes relative to other subcategories, with the biological processes category also showing large numbers relating to cellular processes (9353) and metabolic processes (9039). This suggested an abundance of metabolic activities occurring at the time of sampling.

### 3.4. Functional Classification by KEGG

To further predict the metabolic pathways of* G. sinensis*,* Arabidopsis thaliana* and* Oryza sativa* were used as references and each assembled unigene was annotated in KAAS to obtain the corresponding enzyme commission (EC) numbers. KEGG is considered a basic platform for systematic functional analysis based on constructed networks comprising gene products. KEGG analysis mapped a total of 2914 unigenes to 307 metabolic pathways encompassing five KEGG categories, including metabolism, genetic information processing, environmental information processing, cellular processes, and organismal systems (Additional File 2). The “metabolic” pathways show the highest representation (1357 members, 46.6%), followed by ribosome (180 members, 6.2%, ko03010), biosynthesis of amino acids (147 members, 5.0%, ko01230), carbon metabolism (133 members, 4.6%, ko01200), plant hormone signal transduction (129 members, 4.4%, ko4075), spliceosome (127 members, 4.3%, ko03040), protein processing in the endoplasmic reticulum (125 members, 4.3%, ko04141), purine metabolism (109 members, 3.7%, ko00230), and RNA transport (100 members, 3.5%, ko03013).

### 3.5. Classification by COG

**Figure 4:**
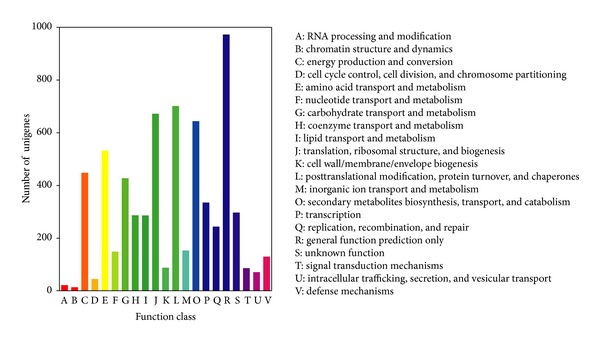
COG function classification of the* G. Sinensis* transcriptome. A total of 6413 unigenes with significant homologies to the COG database (*E*-values ≤ 1.0*E* − 6) were classified into 21 COG categories.

Cluster of Orthologous Groups (COG) database containing proteins encoded by 66 complete genomes of bacteria, algae, and eukaryotes was constructed according to phylogenetic relationships. COG is a very useful tool for predicting the functionality of the individual proteins or all proteins in a new genome. In the present study, all obtained unigenes were compared with proteins in COG and classified into appropriate COG clusters. The results identified 6413 genes displaying significant sequence homology with COG database proteins, accounting for 11.0% of all unigenes. Some unigenes were shown to have multiple COG functions with a total of 6582 functional annotations noted and grouped into 21 COG function sets with *E*-values ≤ 1.0*E* − 6 (Table 2, Figure 4, and Additional File 3). The five sets including the largest number of unigenes were (1) “general function prediction only” (14.8%); (2) “replication, recombination, and repair” (10.6%); (3) “translation, ribosomal structure, and biogenesis” (10.2%); (4) “posttranslational modification, protein turnover, and chaperones” (9.8%); and (5) “amino acid transport and metabolism” (8.1%). The “RNA processing and modification” and “chromatin structure and dynamics” sets contained the least numbers of unigenes, 21 and 13, respectively.

In summary, 31385 unigenes were annotated using NR, Swiss-prot, COG, and KEGG databases with *E*-values ≤ 1.0*E* − 6 deemed significant. Among these unigenes, 1433 showed BLAST match results in all four public databases demonstrating a strong functional annotation. These annotations provide valuable resources for further study of the specific metabolic activities, gene structures, and functions and pathways of* G. sinensis*.

### 3.6. Construction of Protein-Protein Interaction Network in* G. sinensis*

**Figure 5:**
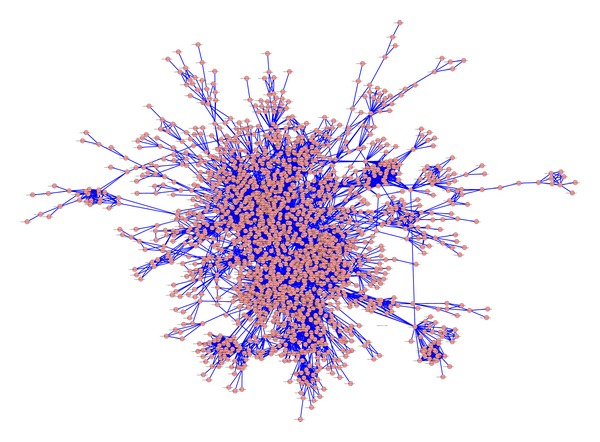
Illustration of the giant component of unigenes in* G. sinensis*.

**Figure 6:**
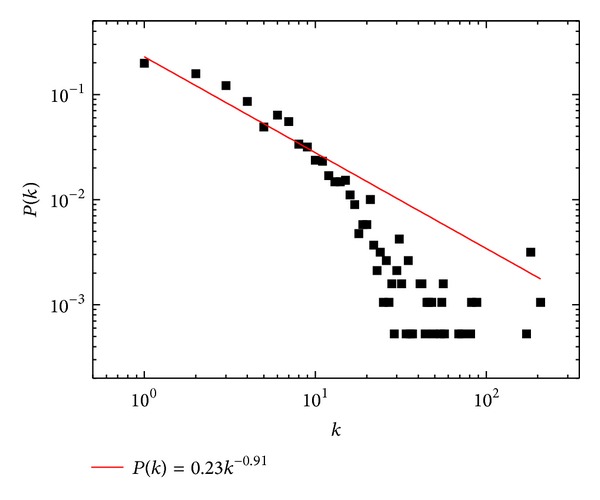
The topological analysis of the giant component in* G. sinensis* with 1897 nodes and 7078 edges. Log-log plots of the node degree distribution with a power-law fit (red line).

Using TBLASTN, similarities between* G. sinensis* unigenes and genes in a PPI network consisting of six String database genomes (*Arabidopsis thaliana*,* Arabidopsis lyrata*,* Oryza sativa* subsp.* japonica*,* Brachypodium distachyon*,* Populus trichocarpa*, and* Sorghum bicolor*) facilitated the construction of a PPI network of* G. sinensis*. This network contained one giant component and 91 smaller components (Figure 5 and Figure S1). The giant component contained 1,897 nodes with 7078 links between nodes. The degree distribution of giant component conformed to *P*(*k*) = 0.23*k* ^−0.91^, implying that the network was scale free and similar to other biological networks (Figure 6).

### 3.7. Prediction of Resistance Genes in* G. sinensis*

Resistance genes and protein sequences from* Arabidopsis thaliana* relating to freezing, drought, and salinity tolerance were downloaded from STIFDB and compared with* G. sinensis* unigenes to locate sequence homology. 435 freezing tolerance genes, 284 drought tolerance genes, and 348 salinity tolerance genes were found (Additional File 4). Based on the constructed* G. sinensis* PPI network, new freezing, drought, and salinity tolerance genes were predicted with a protein considered a potential resistance gene if interactions with over four known resistance genes were noted. The results revealed 19 new freezing tolerance genes, 11 drought tolerance genes, and 18 salinity tolerance genes (Table 3). This provides a theoretical basis for future experimental studies on resistance genes and for culturing resistant* G. sinensis *varieties.

## 4. Discussion

*Gleditsia sinensis* is a tree species of important economic and medicinal value. However, due to a lack of genomic research, molecularly based breeding of* G. sinensis *is hindered. Recently, RNA-seq technology has provided a new approach to obtaining rich sequence information to include successful applications in many plants, such as* Youngia japonica *[29], cabbage [30], tea plant [15], and citrus psyllid [31]. In the present study, RNA-seq was used for transcriptome sequencing of* G. sinensis* with 7.6 Gbp examined and 75.6 million clean reads obtained. The Trinity software was used for* de novo* assembly and a total of 58583 unigenes were obtained. Among these, 31385 unigenes were functionally annotated and shown to participate in a variety of biological processes, accounting for 53.6% of all obtained unigenes.

Besides, freezing, drought, and salinity tolerance genes in* G. sinensis* were predicted by searching for homologous genes linked to resistance and using PPI networks. Currently there are many available methods for gene function prediction based on PPI. For example, George et al. assumed that genes interacting with known disease genes were also disease genes and studied third-degree interactions, yet many false positives were discovered based on nondirect interactions [32]. Xu and Li reported five topological characteristics of disease-related PPI networks, yet these characteristics were not found in the yeast two-hybrid network. These characteristics were used to predict disease-related genes, yet they were unable to explain the biological significance of these characteristics, and their results still required experimental verifications [33]. Based on these previous studies and to ensure the lowest rate of false positive predictions, the present study applied more strict conditions to include direct connections with known resistance genes and interactions with over four known resistance genes. Whether the identified genes are indeed related to resistance still needs further experimental validation and this will be the focus of our future research.

## 5. Conclusion

In the present study, Illumina RNA-seq and* de novo* assembly methods were applied to study the transcriptome of* G. sinensis* for the first time. A total of approximately 75.6 million reads, assembled into 58583 unigenes with an average length of 900 bp, were identified. Among these unigenes, 31385 obtained annotation from NR, Swiss-prot, COG, and KEGG databases. The results of the present study confirm that RNA-seq technology is a fast, effective method for transcriptome analysis of nonmodel plants and provides a good resource for further gene expression analysis. The constructed PPI network for* G. sinensis* when compared to known resistance genes of* Arabidopsis* predicted 18 freezing tolerance genes, 11 drought tolerance genes, and 19 salinity tolerance genes. Thus these findings provide a theoretical basis for future culturing of stress-resistant* G. sinensis* varieties.

## Supplementary Material

Additional File 1: Information for GO classification of *Gleditsia sinensis unigenes*.
Additional File 2: Results for KEGG pathway mapping of *Gleditsia sinensis unigenes*.
Additional File 3: Information for COG classification of *Gleditsia sinensis unigenes*.
Additional File 4: Resistant genes relating to cold, drought and salinity tolerance in *Gleditsia sinensis* identified by comparing to the known resistant genes from *Arabidopsis thaliana*.
Figure S1: Illustration of the PPI network of UniGenes in *G. sinensis*.

## Figures and Tables

**Table 1:** Summary of sequence assembly by Trinity after Illumina sequencing.

	Number	Mean size (bp)	N50 size (bp)	Total nucleotides (bp)
Read	75570488	101	101	7632619288
Unique transcript	142155	1537	1202	218503453
Unigene	58583	900	549	52719022

**Table 2:** Summary of annotation of *G. sinensis* unigenes.

Category	Number	Percentage
NR annotated unigene	31100	53.09%
Swiss-prot	22157	37.82%
GO classified unigene	15264	26.06%
COG classified unigene	6413	10.95%
KEGG classified unigene	2914	4.97%

**Table 3:** Novel resistant genes relating to cold, drought, and salinity tolerance identified by network analysis.

	Cold	Drought	Salinity
comp22423_c0_seq1	Yes	Yes	Yes
comp24732_c0_seq2	Yes	No	No
comp25430_c0_seq1	Yes	No	No
comp28513_c0_seq1	Yes	No	No
comp37332_c0_seq1	Yes	Yes	Yes
comp38200_c0_seq1	Yes	No	No
comp38390_c0_seq5	No	Yes	Yes
comp39646_c0_seq2	Yes	Yes	Yes
comp39900_c0_seq1	No	Yes	Yes
comp39917_c0_seq1	No	No	Yes
comp41481_c0_seq2	Yes	No	No
comp42998_c0_seq1	Yes	No	Yes
comp43037_c0_seq1	Yes	No	Yes
comp43634_c0_seq1	No	Yes	Yes
comp45199_c0_seq1	No	Yes	Yes
comp45561_c0_seq1	Yes	No	No
comp47037_c0_seq1	No	Yes	Yes
comp47415_c0_seq1	Yes	No	Yes
comp47471_c0_seq6	No	Yes	Yes
comp47482_c0_seq2	Yes	No	Yes
comp47503_c0_seq6	Yes	No	Yes
comp47673_c0_seq3	Yes	No	No
comp47694_c0_seq2	Yes	No	No
comp48118_c0_seq4	No	Yes	Yes
comp50179_c0_seq1	Yes	Yes	Yes
comp51180_c1_seq11	Yes	No	Yes
comp81788_c0_seq1	Yes	No	No

## Abbreviations

UT: Unique transcript
NR: NCBI nonredundant protein
COG: Cluster of Orthologous Groups
GO: Gene ontology
KEGG: Kyoto Encyclopedia of Genes and Genomes database
KAAS: KEGG automatic annotation server
BLAST: Basic local alignment search tool
PPI: Protein-protein network.
